# Structural basis of EGF-repeat *O*-glucosylation by the protein *O*-glucosyltransferase POGLUT2

**DOI:** 10.1016/j.jbc.2026.111361

**Published:** 2026-03-09

**Authors:** Yuying Xia, Xinlin Hu, Zhengkang Hua, Min Zhang, Xuyang Ding, Yunshu Shi, Yan Ke, Jiameng Li, Hongjun Yu

**Affiliations:** 1 Department of Biochemistry and Molecular Biology, School of Basic Medicine, Tongji Medical College and State Key Laboratory for Diagnosis and Treatment of Severe Zoonotic Infectious Diseases and Hubei Key Laboratory of Natural Active Polysaccharides, Huazhong University of Science and Technology, Wuhan, China; 2 Department of Pathogen Biology, School of Basic Medicine, Tongji Medical College, Huazhong University of Science and Technology, Wuhan, China; 3 Cell Architecture Research Center, Huazhong University of Science and Technology, Wuhan, China

**Keywords:** POGLUT2, *O*-glucosylation, crystal structure, EGF-repeat, Notch signaling, substrate recognition, disease mutations

## Abstract

Human protein *O*-glucosyltransferase 2 (POGLUT2) catalyzes the *O*-glucosylation of Notch receptors and extracellular matrix proteins, with its dysfunction linked to human disorders. Despite its physiological importance, the structural and mechanistic basis of POGLUT2 has remained elusive. Here, we report the first 1.79 Å structure of POGLUT2 in complex with UDP, revealing a three-domain architecture stabilized by an N-terminal filamin domain, which is unique in Notch-modifying enzymes. Integrated structural, computational, and functional analyses demonstrate that POGLUT2 recognizes structural features within epidermal growth factor–like repeats, including a conserved hydrophobic patch, which explains its stringent substrate selectivity. Our findings further identify Asp238 as the catalytic base, supporting an S_N_2-type inverting mechanism. Furthermore, we show that cancer-associated mutations impair enzymatic activity through distinct structural and mechanistic disruptions. By delineating conserved and divergent features between POGLUT2 and POGLUT1, our study advances the mechanistic understanding of epidermal growth factor–like-repeat *O*-glucosylation and establishes a framework for investigating its dysregulation in human diseases.

The Notch signaling pathway is a fundamental and evolutionarily conserved system that governs cell fate decisions in all metazoans (1, 2, 3). Notch signaling regulates diverse processes, including proliferation, differentiation, and apoptosis (4), and its dysregulation is implicated in developmental disorders and numerous cancers (5, 6). A key regulating feature of the Notch receptor lies in its extensive *O*-linked glycosylation within the Notch extracellular domain, which critically modulates receptor folding, trafficking, and signaling competence (7, 8). Distinct glycosyltransferases (GTs) introduce *O*-glucose, *O*-fucose, and *O*-GlcNAc to defined consensus motifs in epidermal growth factor–like (EGF) repeats of Notch receptors, creating multilayered regulatory codes that fine-tune signaling outcomes (8, 9, 10).

Among these modifications, *O*-glucosylation represents a key regulatory mechanism mediated by the protein *O*-glucosyltransferases (POGLUTs). This enzyme family comprises three members—POGLUT1, POGLUT2 (KDELC1), and POGLUT3 (KDELC2)—that recognize and glucosylate enzyme-specific motifs on Notch EGF-repeat domains (11). These evolutionarily conserved domains (typically comprising ∼30–40 residues) all feature three disulfide bonds formed by six conserved cysteine residues (C^1^–C^6^) (Fig. 1*A*) (12, 13, 14). Specifically, POGLUT1 (known as Rumi in *Drosophila*) modifies serine residues within the C^1^–C^2^ consensus sequence C^1^-X-S-X-(P/A)-C^2^ (with the modified serine underlined) and is essential for Notch signaling and embryonic development (15, 16). In contrast, POGLUT2 and POGLUT3 recognize a distinct C^3^–C^4^ consensus sequence C^3^-X-N-T-X-G-S-F-X-C^4^, thereby targeting a separate set of EGF repeats independently of POGLUT1. *O*-glucosylation by POGLUT2/3 is critical for Notch signaling: suppression of POGLUT3 downregulates the expression of Notch receptors 1 to 3 (17), and loss of *O*-glucose modification impairs Notch activation dependent on Notch-ligand interactions (11). Beyond Notch, recent studies have identified new substrates for POGLUT2/3, including several extracellular matrix (ECM) proteins, such as fibrillin-1, fibrillin-2, and LTBP1, all of which feature tandem EGF repeats (18, 19, 20). Importantly, dysregulation of POGLUT2/3 is closely linked to diseases. Aberrant *O*-glucosylation of fibrillin-1 EGF repeats has been associated with Marfan syndrome (21, 22). Blocking POGLUT2/3-mediated *O*-glucosylation in mice results in neonatal lethality accompanied by skeletal, pulmonary, and ocular defects (23). Furthermore, POGLUT2/3 have also been implicated in various cancers (17, 24, 25, 26, 27).

**Figure 1:**
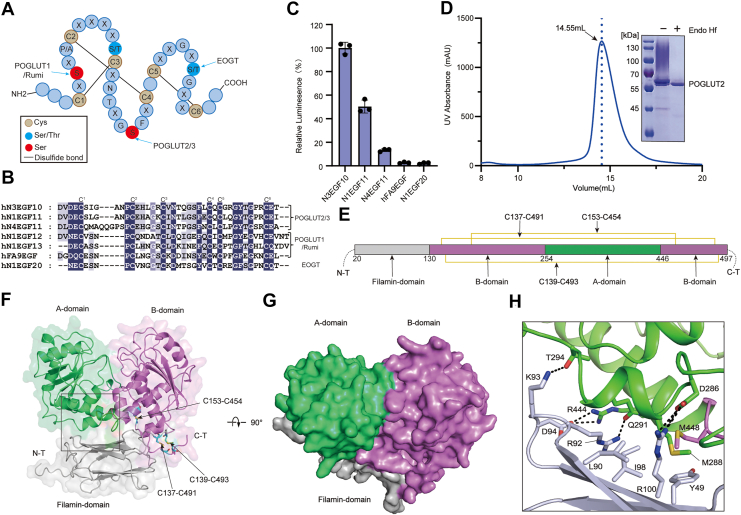
**Functional and structural characterization of human POGLUT2**. *A*, *O*-glucosylation sites within epidermal growth factor–like (EGF) repeats. POGLUT1 (Rumi in *Drosophila*) adds *O*-Glc to serine within the C^1^–C^2^ consensus sequence C^1^-X-S-X-(P/A)-C^2^. POGLUT2/3 adds *O*-Glc to serine residue within the C^3^–C^4^ consensus sequence C^3^-X-N-T-X-G-S-F-X-C^4^. EOGT (EGF domain–specific *O*-GlcNAc transferase) catalyzes *O*-GlcNAc addition to serine or threonine within the C^5^–C^6^ consensus sequence C^5^-X-X-G-X-(S/T)-G-X-X-C^6^. Six conserved cysteine residues (C1–C6) form three disulfide bonds, indicated by *solid lines*. *B*, sequence alignment of representative EGF-repeat substrates of POGLUT2/3 (human Notch3 EGF10, hN3EGF10; human Notch1 EGF11, hN1EGF11; human Notch4 EGF11, hN4EGF11), POGLUT1 (human Notch1 EGF12 and 13, hN1EGF12 and hN1EGF13; human factor IX EGF, hFA9EGF) and EOGT (human Notch1 EGF20, hN1EGF20). *C*, enzymatic activity of POGLUT2 with different EGF repeats as acceptor substrates (hN3EGF10, hN1EGF11, and hN4EGF11). hFA9EGF (POGLUT1 substrate) and hN1EGF20 (EOGT substrate) serve as negative control. Data represent mean ± SD from three independent assays. *D*, preparation of homogeneous POGLUT2. SDS-PAGE analysis shows removal of heterogeneous *N*-glycans by Endo Hf digestion. The resulting deglycosylated POGLUT2 was further purified by gel-filtration chromatography (Superdex 200 Increase 10/300 GL). *E*, schematic of the domain organization of POGLUT2. Three pairs of disulfide bonds are depicted by *yellow lines*. *F*, overall structure of POGLUT2. *Cartoon representation* highlights A-domain (*green*), B-domain (*pink*), and filamin domain (*dark gray*). The disulfide bonds are depicted as *cyan sticks*. *G*, surface representation of POGLUT2 in the *top view*. *H*, close-up view of interdomain interactions between the filamin domain and A-domain, as indicated by the *box* in (*F*). POGLUT2, protein O-glucosyltransferase 2.

POGLUT2 and POGLUT3 are close homologs within the GT90 family of GTs and feature a unique N-terminal filamin domain absent in other Notch-modifying GTs. They transfer glucose from UDP-α-D-glucose to serine residues within the C^3^–C^4^ consensus sequence of EGF repeats, generating a β-linked *O*-glucosylation (Fig. 1*A*) (19). Despite their biological importance, key aspects, such as their 3D structures, substrate recognition, catalytic mechanism, and even the precise location of their active sites, remain poorly understood. Indeed, molecular insights into protein-modifying GTs remain limited (28), with only a few examples—such as POGLUT1 (15, 16), POFUT1 (29), and POFUT2 (30)—having been characterized to date. The latter two (POFUT1 and POFUT2) are protein *O*-fucosyltransferases that transfer fucose residues from GDP-fucose to specific serine/threonine residues within EGF repeats or thrombospondin type-1 repeats. Markedly, POGLUT2/3 share high sequence homology (∼30.6% identity) with POGLUT1; the molecular basis for their recognition of distinct consensus sequences within EGF repeats remains elusive (31).

In this study, we report the first crystal structure of human POGLUT2 in complex with UDP. The structure reveals a three-domain architecture unique to Notch-modifying enzymes and delineates the molecular determinants for ligand binding and catalytic activity. Combining structural, computational, and functional analyses, we elucidate the molecular mechanism of EGF-repeat *O*-glucosylation by POGLUT2. Furthermore, we provide mechanistic insights into how cancer-associated mutations impair the enzyme’s function, highlighting the relevance of POGLUT2’s catalytic mechanism in human diseases.

## Results

### Functional characterization of POGLUT2

POGLUT2 has been proposed to glycosylate serine residues within the consensus motif C^3^-X-N-T-X-G-S-F-X-C^4^ (where X denotes any amino acid) (Fig. 1*A*), and C^3^ and C^4^ are the third and fourth conserved cysteines within the EGF repeat. Sequence alignment of multiple Notch EGF domains (Fig. 1*B*) revealed a conserved six-cysteine pattern forming three disulfide bonds (C^1^–C^3^, C^2^–C^4^, and C^5^–C^6^) that stabilize EGF-repeat 3D structure (Fig. 1*A*). To assess its substrate specificity, we performed *in vitro* GT assays using purified human POGLUT2 and three EGF repeat substrates derived from different human Notch paralogs—Notch3 EGF10, Notch1 EGF11, and Notch4 EGF11 (11). To further examine the selectivity of POGLUT2, we included two negative control substrates (Fig. 1*B*): the human factor IX EGF repeat, which contains the POGLUT1-preferred C^1^-X-S-X-(P/A)-C^2^ motif (15), and Notch1 EGF20 containing the EGF domain–specific *O*-GlcNAc transferase–preferred C^5^-X-X-G-X-(S/T)-G-X-X-C^6^ motif (EOGT) (32).

UDP-Glo-based glycosylation assays revealed that POGLUT2 displays distinct substrate preferences (Fig. 1*C*). Among the test substrates, POGLUT2 exhibits the highest activity toward Notch3 EGF10. Notch1 EGF11 was glycosylated with moderate efficiency (∼50%), whereas activity toward Notch4 EGF11 was markedly reduced (∼15%). As expected, POGLUT2 failed to glycosylate human factor IX EGF repeat and Notch1 EGF20, indicating that it does not act on the POGLUT1- or EGF domain–specific *O*-GlcNAc transferase–recognized motifs. These results confirm that POGLUT2 recognizes a distinct consensus motif and can discriminate among closely related EGF domains, highlighting its substrate selectivity based on subtle sequence variations within the EGF repeat scaffold.

### Overall structure of POGLUT2

To gain molecular insights into POGLUT2, we carried out a structural study of the human enzyme. We successfully expressed and purified a highly pure (>95%) and homogeneous form of human POGLUT2, lacking both its N-terminal signal peptide and C-terminal KDEL retention motif, using the *Pichia pastoris* system (Fig. 1*D*). Initial attempts to crystallize the apo form of POGLUT2, however, did not yield well-diffracting crystals. Fortunately, high-quality crystals were obtained when POGLUT2 was cocrystallized with donor substrate UDP-glucose. Due to hydrolysis during the crystallization process, only the UDP moiety remained bound in the active site. Consequently, we determined the structure of the POGLUT2–UDP binary complex at 1.79 Å resolution (Table S1).

The structure resolves residues 20 to 497 of POGLUT2, revealing a three-domain architecture comprising an A-domain, a B-domain, and an N-terminal filamin domain (Fig. 1, *E* and *F*, Fig. S2). The A-domain (residues 254–446) features a central β-sheet (β15–β18) flanked by multiple α-helices (Fig. S1*A*), forming a Rossmann-like fold topology. The B-domain is formed by two discontinuous segments in the primary sequence (residues 130–253 and 447–497), which assemble into a second Rossmann-like fold (Fig.1*E*, Fig. S2*B*). The integrity of the B-domain is reinforced by three interfragment disulfide bonds (C137–C491, C139–C493, and C153–C454), providing structural stabilization (*E* and *F*). The A- and B-domains are arranged in a face-to-face orientation (with a buried surface area of ∼1275 Å^2^) (Fig. 1*G*), forming a canonical glycosyltransferase-B (GT-B) fold (33). This creates a deep cleft likely accommodating the donor and acceptor substrates (see below).

Notably, POGLUT2 possesses an N-terminal filamin domain, which, to our knowledge, is absent in previously characterized GT-Bs. This domain connects to the B-domain and forms extensive stabilizing contacts with the A-domain, including multiple salt bridges (R444–D94, D286–R100), hydrogen bonds (K93–T294, R92–Q291), and hydrophobic interactions (L90–I98, M448–M288–Y49 cluster) (Fig. 1*H*). This multivalent contact network distinguishes POGLUT2 from other GT-B enzymes and stabilizes the overall three-domain architecture of POGLUT2. Consistent with this, structural comparison of the POGLUT2 filamin domain with a previously reported NMR structure of this domain (Protein Data Bank ID: 2DI7) yielded an RMSD of 1.39 Å over 96 aligned Cα atoms, with major deviations localized at the interface with the A-domain, indicating active interdomain interactions within the full-length enzyme (Fig. S3*A*). To experimentally assess the functional role of the filamin domain, we generated a truncated POGLUT2 construct lacking this domain (ΔFilamin). In contrast to WT POGLUT2, the truncated variant failed to produce detectable soluble expression (Fig. S3*B*), supporting that the filamin domain is involved in maintaining structural stability.

### The binding of the donor ligand in human POGLUT2

**Figure 2:**
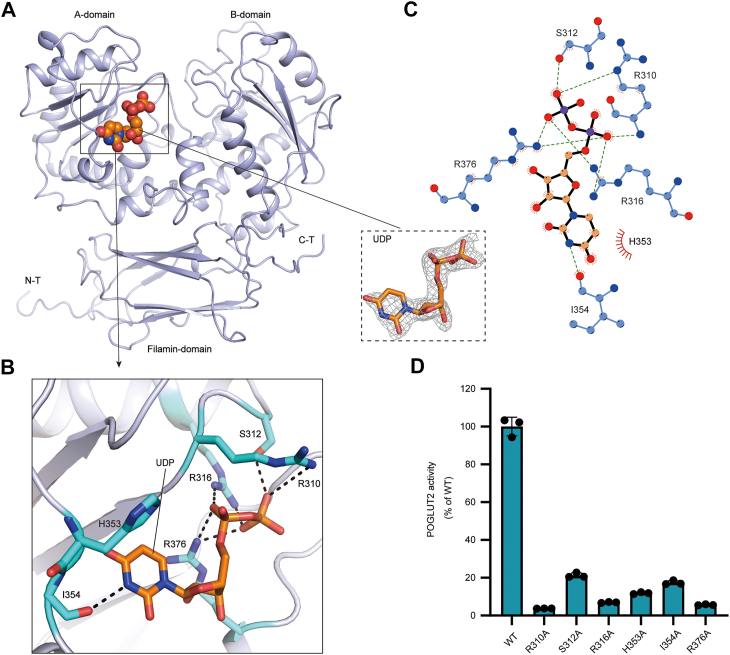
**Ligand binding in POGLUT2**. *A*, structure of POGLUT2 (*blue cartoon*) with UDP ligand (*orange sphere*) bound to the A-domain. *Inset*, modeled UDP (*orange stick*) superimposed with its corresponding electron density (*gray mesh*). The *inset* displays a 2Fo–Fc electron density map surrounding the bound UDP molecule, contoured at 1.5 σ. *B*, close-up view of the UDP binding pocket in the A-domain, showing residues involved in ligand binding (*blue sticks*). Hydrogen bonds and salt bridges are indicated by *dashed lines*. *C*, LIGPLOT + schematic of the UDP interaction network. *D*, enzymatic activity of POGLUT2 mutants at UDP-binding sites, with WT included for comparison. Data represent mean ± SD from three independent assays. POGLUT2, protein *O*-glucosyltransferase 2.

As we described above, we obtained high-quality crystals by cocrystallizing POGLUT2 with donor UDP-glucose. A well-defined electron density peak was observed at the base of the interdomain cleft corresponding unambiguously to bound UDP (Fig. 2*A*, *inset*), indicating that UDP-glucose underwent hydrolysis during crystallization. The bound UDP makes contact with multiple conserved POGLUT2 residues from the A-domain (Fig. 2, *B* and *C*). Specifically, the uracil moiety is nestled within a hydrophobic pocket formed by H353 and I354, whereas the ribose engages in a polar interaction with R376. The diphosphate group is anchored *via* salt bridges to surrounding charged residues R310, R316, and R376 and further stabilized by the side chain of S312. As we were unable to obtain the structure of apo-POGLUT2, we compared our UDP-bound POGLUT2 with the AlphaFold-predicted apo-POGLUT2 structure. Their superposition yielded an RMSD of 0.51 Å, indicating that the UDP binding does not induce significant conformational changes in the overall structure and the catalytic center (Fig. S3*C*).

To probe the functional importance of these donor-related interactions, we performed structure-guided mutagenesis of the UDP-binding site, followed by enzymatic assays. As a result, substitution of R310, R316, or R376 with alanine severely impaired the activity (<5%), underscoring their critical roles in diphosphate coordination and catalysis (see below) (Fig. 2*D*, Fig. S4*A*). Moreover, alterations to residues contacting the uracil base (H353A, I354A) or the beta-phosphate group (S312A) also led to marked reductions in enzymatic activity (<20%), collectively highlighting the importance of precise donor recognition across all parts of the molecule.

### Molecular basis of acceptor EGF-repeat recognition

**Figure 3:**
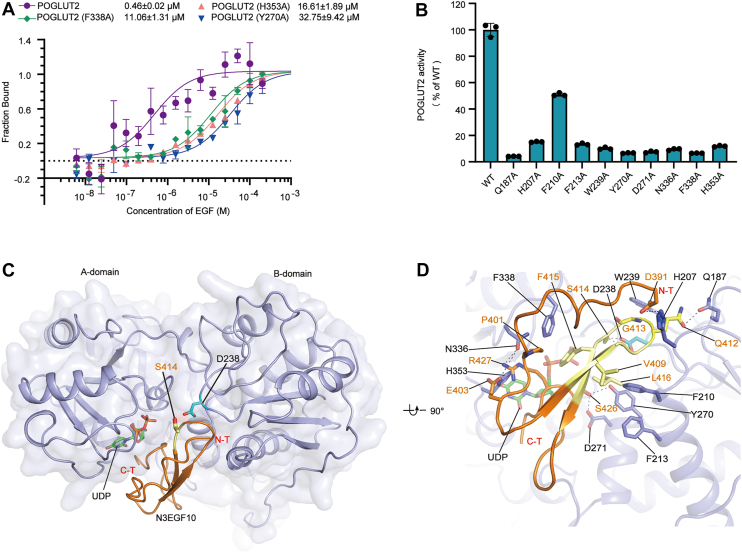
**EGF-repeat binding in POGLUT2**. *A*, binding affinity of hNotch3 EGF10 (N3EGF10) to POGLUT2 and its mutants at the EGF-repeat binding interface, measured by the microscale thermophoresis (MST) assay. The *K*_*d*_ values are provided; data represent mean values ± SDs from three independent experiments. *B*, model of the POGLUT2–UDP–N3EGF10 complex generated by docking hNotch3 EGF10 (N3EGF10) into the POGLUT2–UDP structure. POGLUT2 is shown as a *blue cartoon*, N3EGF10 as an *orange cartoon*, UDP as *green sticks*, S414 of N3EGF10 as a *yellow stick*, and D238 of POGLUT2 as a *cyan stick*. *C*, close-up view of the interface between POGLUT2 and EGF repeat as revealed in (*B*). The C^3^–C^4^ consensus sequence C^3^-X-N-T-X-G-S-F-X-C^4^ (highlighted in *yellow*) inserts into the binding cleft, with the acceptor serine (S414) positioned near the donor diphosphate group and the general base (D238). *D*, enzymatic activity of POGLUT2 mutants at the EGF-repeat binding interface. Data represent mean ± SD from three independent assays. EGF, epidermal growth factor–like; POGLUT2, protein *O*-glucosyltransferase 2.

Despite extensive crystallization trials under diverse conditions, we were unable to obtain cocrystals of POGLUT2 with its EGF substrates (hNotch3 EGF10, hNotch1 EGF11, and hNotch4 EGF11), all of which are confirmed to be enzymatically competent (Fig. 1*C*). This is likely because of the moderate affinity of their interactions, with even the preferred substrate, Notch3 EGF10, binding at a dissociation constant (*K*_*d*_) of 0.46 μM (Fig. 3*A*). To circumvent this limitation and gain mechanistic insights into acceptor substrate recognition, we employed a structure-guided docking approach, integrating with extensive biochemical validation. Using our 1.79 Å structure of POGLUT2–UDP binary complex as the template, we generated a structural model of the ternary POGLUT2–Notch3 EGF10–UDP complex *via* ClusPro 2.0 (Fig. 3*B*) (34, 35). The docking solutions converged on a single, energetically favorable pose, in which the hNotch3 EGF10 binds within the interdomain cleft between A- and B-domains of POGLUT2, engaging in extensive protein–protein interactions. Critically, the consensus motif (C^3^-X-N-T-X-G-S-F-X-C^4^), forming a U-shaped structure comprising two antiparallel β-strands connected by a sharp turn, is nestled deep within the binding cleft. In this configuration, the acceptor Ser414 is positioned in close proximity to the β-phosphate of UDP in the active site, appropriate for glucose transfer, supporting the reliability of this docking result.

Substrate recognition appears to be driven primarily by the consensus motif, which engages POGLUT2 through a combination of hydrophobic and polar interactions (Fig. 3*C*): notable hydrophobic contacts include V409 (subsite −5, with the modified serine defined as subsite 0) with POGLUT2 F210, G413 (subsite −1) with POGLUT2 W239, F415 (subsite +1) with POGLUT2 F338, and L416 (subsite +2) with POGLUT2 F210, Y270 and F213; in parallel, Q412 (subsite −2) forms polar interactions with POGLUT2 Q187. Additional contacts are made by the N-terminal region of the EGF repeat—D391, P401, and E403 interacting with POGLUT2 H207, F338, and H353, respectively—as well as the C-terminal region, where S426 and R427 engage POGLUT2 D271 and N336.

To experimentally validate the model, we performed alanine-scanning mutagenesis of POGLUT2 residues lining the putative substrate-binding interface and measured their enzymatic activity (Fig. 3*D*, Fig. S4*B*). While only F210A had a modest effect, nine of 10 mutants showed substantial loss of activity, underscoring the functional importance of these residues. We further quantified the contribution of selected residues to substrate binding using microscale thermophoresis (MST) (Fig. 3*A*). Mutations such as F338A, H353A, and Y270A caused 20- to 75-fold increases in *K*_*d*_ relative to WT POGLUT2, consistent with severely impaired substrate binding. Collectively, the strong correlation between catalytic activity and binding affinity supports the validity of our docking model and affirms the essential contribution of these residues to substrate engagement.

### Divergent substrate-binding modes with a shared recognition signature by POGLUT2 and POGLUT1

**Figure 4:**
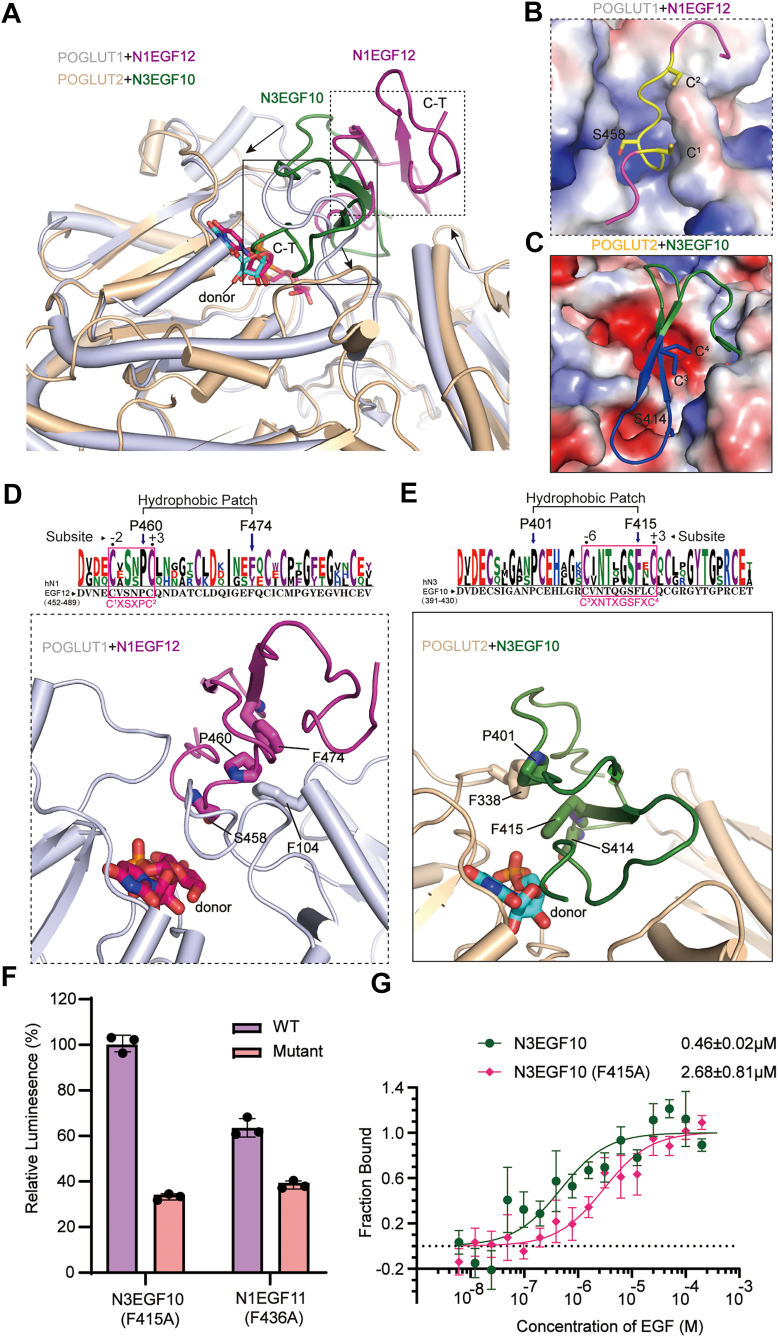
**Recognition of a conserved hydrophobic patch of EGF repeats by POGLUT2 and POGLUT1**. *A*, structural superposition between N3EGF10-bound POGLUT2 (as modeled in Fig. 3*B*) and N1EGF12-bound POGLUT1 (Protein Data Bank ID: 5L0R). *Arrows* indicate interface loop rearrangements from POGLUT1 to POGLUT2, accommodating divergent substrate-binding modes. *B*, detailed view of C^1^–C^2^ consensus sequence (highlighted in *yellow*) of EGF repeat bound to POGLUT1 (in electrostatic surface representation). For clarity, only the region of the EGF repeat near the consensus sequence is displayed. *C*, detailed view of C^3^–C^4^ consensus sequence (highlighted in *blue*) of the EGF repeat bound to POGLUT2 (in electrostatic surface representation). For clarity, only the region of EGF repeat near the consensus sequence is displayed. *D*, close-up view of the EGF-repeat binding interface of POGLUT1, as indicated by the *dashed box* in *A*. The conserved hydrophobic patch (P460 and F474) forms stacking interactions with F104 of POGLUT1. *E*, close-up view of the EGF-repeat binding interface of POGLUT2, as indicated by the *solid box* in *A*. The conserved hydrophobic patch (P401 and F415) engages in stacking with F338 of POGLUT2. *D* and *E*, the corresponding sequence logo analyses of EGF repeats are shown above the structures. *F*, enzymatic activity of POGLUT2 toward EGF-repeat mutants of the conserved hydrophobic patch (N3EGF10 F415A; N1EGF11 F436A). Data represent mean ± SD from three independent assays. *G*, binding affinity of N3EGF10 and its hydrophobic patch mutant F415A to POGLUT2, measured by the microscale thermophoresis (MST) assay. The *K*_*d*_ values are provided; data represent mean values ± SDs from three independent experiments. EGF, epidermal growth factor–like; POGLUT, protein *O*-glucosyltransferase.

POGLUT2 and POGLUT1 catalyze *O*-glucosylation on distinct serine residues embedded within different EGF consensus motifs (Figs. 4*A* and 1*A*) (11). Despite their overall sequence identity (30.6%) and the presence of a unique filamin domain in POGLUT2 (Fig. S1*B*), both enzymes share a conserved GT-B fold consisting of dual Rossmann-like domains, with an RMSD of 1.028 Å over 233 aligned Cα atoms. Notably, structural alignment reveals that the bound EGF repeats are rotated by ∼90° relative to each other and that the substrate-binding surfaces of the two enzymes have adapted accordingly to enforce strict motif specificity (Fig. 4*A*). POGLUT1 features a narrow cleft, optimized for the slender loop of the C^1^-X-S-X-(P/A)-C^2^ consensus motif (Fig. 4*B*). By contrast, POGLUT2 features a wider cleft shaped by shortened or rearranged surface loops (residues 204–209, residues 278–288, and residues 334–341), allowing accommodation of the bulkier U-turn structure of the C^3^-X-N-T-X-G-S-F-X-C^4^ motif (Fig. 4, *A* and *C*).

Despite these divergent binding modes, both enzymes engage a shared structural feature in their acceptor substrates—a conserved hydrophobic patch comprising a proline and an aromatic residue (phenylalanine or tyrosine), brought into proximity by the EGF fold. In POGLUT1 (Rumi), this Pro-Phe/Tyr patch (P460 and F474 in Notch1 EGF12) is stabilized by F104 from the B-domain (Fig. 4*D*) (15, 16). In the POGLUT2 ternary complex model, the rotated orientation of the EGF repeat positions a similar patch (P401 and F415 in Notch3 EGF10) against F338 of the A-domain (Fig. 4*E*). WebLogo analysis reveals high conservation of hydrophobic phenylalanine and proline residues within POGLUT2-recognized EGF substrates. This hydrophobic interaction is functionally critical: mutation of F338 to alanine markedly reduced POGLUT2 activity (Fig. 3*D*). Consistently, alanine substitution of the conserved phenylalanine in the substrate (F415A in Notch3 EGF10 or F436A in Notch1 EGF11) significantly impaired enzymatic activity (Fig. 4*F*). MST further demonstrated that the F415A mutation dramatically weakened binding affinity (Fig. 4*G*), establishing this conserved Pro–Phe/Tyr patch as a key determinant of substrate recognition by POGLUT2.

### The catalytic mechanism of POGLUT2

**Figure 5:**
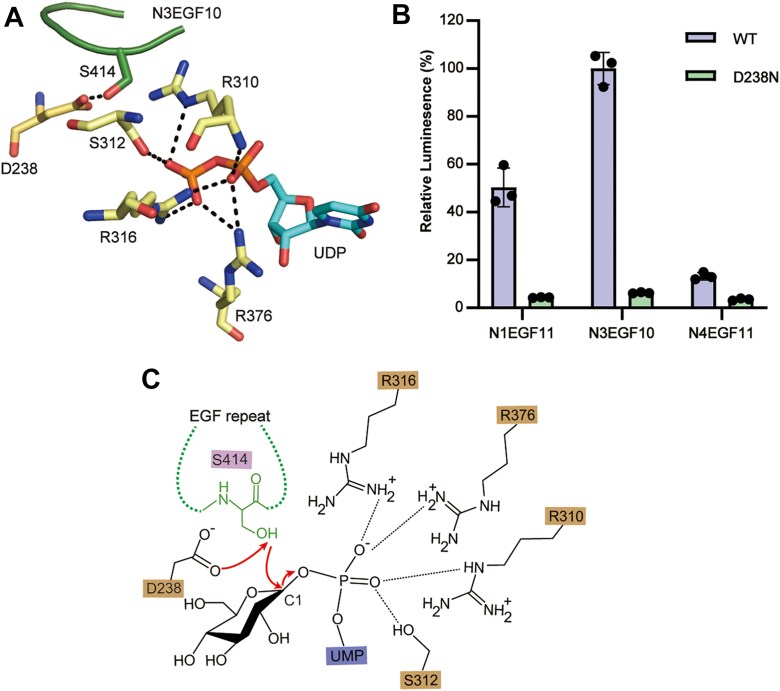
**Reaction mechanism of POGLUT2**. *A*, close-up view of the active site in the POGLUT2–UDP–N3EGF10 complex (as modeled in Fig. 3*B*). *Dashed lines* indicate hydrogen bonds. POGLUT2 D238 is positioned near the acceptor serine (S414). Enzyme residues involved in diphosphate binding are shown in *stick representation*. *B*, enzymatic activity of WT POGLUT2 and the catalytic mutant D238N toward three EGF repeats (N1EGF11, N3EGF10, and N4EGF11). Data represent mean ± SD from three independent assays. *C*, proposed reaction mechanism of *O*-glucosylation by POGLUT2. POGLUT2 D238 acts as the catalytic base to deprotonate the serine hydroxyl group of EGF repeats, whereas POGLUT2 R310, R316, and R376 facilitate the departure of the UDP-leaving group. EGF, epidermal growth factor–like; POGLUT2, protein *O*-glucosyltransferase 2.

The POGLUT2 ternary complex model offers key insights into its catalytic mechanism. Structural inspection of the active site reveals that D238, an invariant residue, is positioned within 4 Å of the acceptor serine (S414), ideally placed to function as the catalytic base by activating the nucleophilic hydroxyl group (Fig. 5*A*). In parallel, the UDP diphosphate is coordinated by three conserved arginine residues (R310, R316, and R376), which likely stabilize the developing negative charge and facilitate the departure of the leaving group. This configuration is consistent with an S_N_2-type inverting mechanism (36), similar to those described for other GTs, such as POMGnT1 (37), POGLUT1 (15, 16), and POFUT1 (29). To validate the catalytic role of D238, we generated a D238N mutant, which retains side-chain size and geometry but lacks general base functionality. As anticipated, the D238N variant exhibited nearly abolished enzymatic activity (Fig. 5*B*, Fig. S4, *C* and *E*). This result establishes D238 as essential for catalysis and supports the conclusion that POGLUT2 operates *via* an S_N_2-like inverting mechanism, in line with other GT90 family GTs (Fig. 5*C*).

### Cancer-associated mutations impair POGLUT2 activity

**Figure 6:**
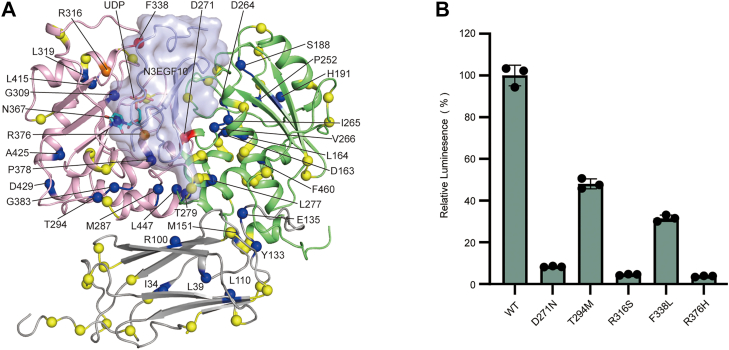
**Cancer-related mutations in POGLUT2 adversely affect enzymatic activity**. *A*, the cancer-associated POGLUT2 mutations were mapped onto the model of POGLUT2–UDP–N3EGF10 complex. These mutants were identified from cBioPortal (http://www.cbioportal.org/). The EGF repeat and UDP are colored *light blue* and *cyan*, respectively. The POGLUT2 A-domain is in *pink*, the B-domain in *light green*, and the filamin domain in *gray*. The mutated residues are depicted as *spheres* and highlighted by functional region: *red*, EGF-repeat binding; *orange*, donor binding; *blue*, buried within the structure; *yellow*, surface exposed. *B*, effects of POGLUT2 cancer-related mutations on *in vitro* enzymatic activity. Data represent mean ± SD from three independent assays. EGF, epidermal growth factor–like; POGLUT2, protein *O*-glucosyltransferase 2.

We have previously found that the dysfunction of Notch-modifying GT is linked to human cancers (38, 39, 40). Given POGLUT2’s critical role in modulating Notch signaling—a pathway central to cancer—we investigated whether POGLUT2 is altered in human tumors. Analysis of cancer genomics data *via* cBioPortal revealed frequent POGLUT2 alterations across various cancer types (41, 42). Notably, high-level gene amplifications were recurrent in lung squamous cell carcinoma, esophageal carcinoma, and endometrial carcinoma. Beyond copy number changes, numerous somatic missense mutations were identified. Mapping these onto our POGLUT2 structure revealed nonrandom clustering in four key regions: the UDP-binding pocket, the predicted EGF-binding cleft, the critical intramolecular interaction region, and the surface-exposed sites (Fig. 6*A*). Among the identified point mutations, we selected two representative variants from each of the first three critical regions and characterized them *in vitro*; mutations located at the surface-exposed sites were not tested as they are predicted to result in relatively mild functional effects. Mutations targeting the catalytic arginines (R316S, R376H) completely abolished enzymatic activity, consistent with their role in UDP stabilization (Fig. 6*B*, Fig. S4*D*). D271N, located at the modeled EGF-binding interface, reduced activity to ∼10% of WT, supporting its importance in substrate recognition. Hydrophobic core mutation F338L, which also contacts the substrate, had a milder impact. Mutations affecting the intramolecular interaction region also varied in severity: T294M retained more moderate activity (∼50% activity), whereas D429N severely impaired activity, showing position-specific effects. These findings establish a mechanistic link between cancer-associated POGLUT2 mutations and loss of enzymatic function. They suggest that in tumors where Notch signaling is tumor suppressive, impaired POGLUT2 activity may contribute to oncogenesis through dysregulated glycosylation of Notch receptors.

## Discussion

This study defines the structural and mechanistic basis of human POGLUT2, a GT critical for the modification and regulation of Notch receptors and ECM proteins. We present the first structure of POGLUT2 complexed with UDP, revealing a distinctive three-domain organization. Notably, a unique N-terminal filamin domain, absent in previously characterized GTs, stabilizes the canonical GT-B fold formed by the A- and B-domains.

We further demonstrate that POGLUT2 exhibits strict substrate selectivity for EGF repeats harboring the C^3^–C^4^ consensus sequence C^3^-X-N-T-X-G-S-F-X-C^4^ motif. Combined computational modeling, mutagenesis, and functional assays suggest that POGLUT2 recognizes 3D structural features of EGF repeats instead of this linear consensus sequence. More importantly, we identified a conserved hydrophobic patch, formed by a phenylalanine residue at subsite +1 and a proline residue outside the motif, which serves as a critical structural determinant for recognition (Fig. 4, *C* and *E*). This element, broadly conserved across EGF repeats, is also utilized by POGLUT1 to recognize the distinct C^1^–C^2^ consensus sequence (C^1^-X-S-X-(P/A)-C^2^), albeit through a fundamentally different binding mode (Fig. 4, *B* and *D*) (43). Notably, this same hydrophobic patch has previously been implicated in stabilizing the Glc-Xyl-Xyl trisaccharide modification, underscoring its broader role as a structural signature in the regulation of EGF-repeat glycosylation (8).

Our study also clarifies the catalytic mechanism of POGLUT2. The donor diphosphate is stabilized by three conserved arginine residues (R310, R316, and R376), whereas D238 is ideally positioned to act as the catalytic base by deprotonating the acceptor serine (Fig. 2). Consistently, mutation of D238 to asparagine nearly abolished enzymatic activity, supporting this residue as essential for catalysis (Fig. 5*B*). Together, these features support an S_N_2-like inverting mechanism, in line with other GT90 family GTs (15, 16).

Our results also provide a mechanistic framework for interpreting disease-associated mutations. Structural and biochemical analyses suggest how POGLUT2 alterations may disrupt substrate recognition, catalysis, or enzyme stability, potentially contributing to cancer pathogenesis. Indeed, dysfunction of other EGF-repeat–modifying GTs, such as XXYLT1 and POGLUT1 (Rumi), has been linked to human cancers (44, 45). Given their ability to modify both Notch and ECM proteins, these findings underscore their broader significance in tumor biology and support further exploration of their therapeutic potential—particularly in light of their high specificity (46, 47, 48), an area that warrants further investigation.

In summary, this study provides mechanistic insights into POGLUT2, including its molecular architecture, substrate recognition, catalytic chemistry, and disease relevance. By delineating both conserved and divergent principles relative to POGLUT1, our work establishes a molecular foundation for understanding *O*-glucosylation of EGF repeats and for exploring the pathogenic role of glucosylation dysfunction in human diseases.

## Experimental procedures

### Cloning, expression, and purification of human POGLUT2

The DNA sequence of human POGLUT2 was amplified from the complementary DNA of 293T cells. The POGLUT2, encompassing residues 20 to 502, was cloned into the pPICZαA vector (Invitrogen) with a C-terminal His_6_-tag. The recombinant plasmids were linearized by the restriction enzyme PmeI and transformed into *P*. *pastoris* strain X33 by electroporation. After selection, transformants were cultured in BMGY medium and induced in BMMY medium at 28 °C, according to the EasySelect Pichia Expression Kit manual (Invitrogen).

For purification of secreted POGLUT2, the supernatant collected from a 3 l culture was subjected to nickel–nitrilotriacetic acid affinity chromatography (GE Healthcare). After washing with buffer A (20 mM Tris–HCl, 300 mM NaCl, pH 7.5), the target protein was eluted with buffer B (20 mM Tris–HCl, 300 mM NaCl, 500 mM imidazole, pH 7.5). The eluate was desalted using a HiPrep 26/10 Desalting Column (GE Healthcare). To remove *N*-glycans, the target protein was incubated with maltose-binding protein (MBP)–tagged Endo Hf (NEB) at a 1:100 (w/w) enzyme-to-protein ratio overnight at 4 °C. After digestion, the MBP-tagged Endo Hf was removed using the MBP Trap HP column (GE Healthcare). The treated target protein was concentrated and further purified by size-exclusion chromatography on a Superdex 200 10/300 GL column (GE Healthcare) pre-equilibrated in buffer C (20 mM Tris–HCl, 150 mM NaCl, pH 7.5). The central fractions corresponding to the monodisperse peak were pooled and concentrated to 7 mg/ml for subsequent applications. Purity of the final sample was verified by SDS-PAGE (10% polyacrylamide gel) under reducing conditions, followed by Coomassie Brilliant Blue R-250 staining and destaining with 10% acetic acid and 40% methanol.

### Cloning, expression, and purification of EGFs

DNA sequences encoding human Notch1 EGF11, Notch3 EGF10, Notch4 EGF11, and Notch1 EGF20 were codon-optimized for expression in *Escherichia coli* and synthesized commercially (GenScript). The resulting DNA fragments were cloned into a pET32a vector containing an N-terminal TrxA-His_6_ tag and a human rhinovirus 3C protease cleavage site.

Preparation of EGF repeats was performed as previously described (49, 50). Specifically, recombinant plasmids were transformed into BL21 (DE3) *E*. *coli* and cultured in LB medium at 37 °C until the absorbance reached approximately 0.6 at 600 nm. Protein expression was induced by adding 0.5 mM IPTG, followed by incubation at 16 °C for 16 h. Cells were harvested and lysed by French press in lysis buffer (20 mM Tris–HCl, 300 mM NaCl, pH 7.5) supplemented with protease inhibitors and 1 mM PMSF. After centrifugation at 13,000*g* for 1 h, the supernatant was applied to a nickel–nitrilotriacetic acid affinity column. After washing, the target protein was eluted with elution buffer (20 mM Tris–HCl, 300 mM NaCl, 500 mM imidazole, pH 7.5). To remove the N-terminal tag, the eluted protein was incubated with human rhinovirus 3C protease at a 1:150 (w/w) enzyme-to-protein ratio at 4 °C for 16 h. The digested protein was then concentrated and further purified by size-exclusion chromatography using a Superdex 75 10/300 GL column pre-equilibrated with 20 mM Tris–HCl, 150 mM NaCl, pH 7.5. Fractions containing the EGF repeats were pooled and concentrated to 4 mg/ml for subsequent use.

### Crystallization and ligand soaking

For POGLUT2 crystallization, the purified protein at a concentration of 7 mg/ml was used in initial screening and subsequent reproduction of crystals. Crystals were grown at 20 °C using the hanging-drop vapor diffusion method. The optimized reservoir solution contained 0.2 to 0.3 M sodium formate (pH 7.2) and 20% to 30% (w/v) polyethylene glycol 3350.

For ligand soaking in the POGLUT2 crystals, UDP-glucose was added to the crystal-containing drops to a final concentration of 15 mM and allowed to soak for 30 to 120 s prior to harvesting. The crystals were then flash-frozen in liquid nitrogen. Both POGLUT2 crystals and the ligand-soaked crystals were cryoprotected by brief immersion in a solution consisting of the reservoir solution supplemented with 30% (v/v) glycerol before freezing.

### X-ray data collection, structure determination, and molecular docking

All X-ray diffraction datasets were collected at beamline BL19U1 of the Shanghai Synchrotron Radiation Facility using a wavelength of 0.9791 Å. Diffraction images were processed, merged, and scaled using XDS (https://xds.mr.mpg.de/index.html) and the CCP4 suite (https://www.ccp4.ac.uk/). The native and ligand-soaked crystals had a space group of *P 1 21 1* with one protein molecule in the asymmetrical unit.

The structure of POGLUT2 was determined by molecular replacement using Phaser from the PHENIX suite (https://www.phenix-online.org/), with an AlphaFold2-generated model serving as the search template. The resulting initial model was subjected to automatic refinement in PHENIX, followed by manual rebuilding in Coot (https://www2.mrc-lmb.cam.ac.uk/personal/pemsley/coot/) and several rounds of refinement in PHENIX with secondary structure and geometric restraints. The UDP-bound POGLUT2 complex structure was determined following a similar strategy: the initial model without ligand was determined using molecular replacement, followed by iterative cycles of refinement in PHENIX and manual rebuilding in Coot. Then the *Fo−Fc* difference maps and the *2Fo−Fc* electron density maps were carefully analyzed before building the ligand into the density map. Detailed data collection and structure refinement statistics are provided in Table S1.

To investigate the molecular basis of EGF-repeat substrate recognition by POGLUT2, computational protein–protein docking was performed using the structure of POGLUT2–UDP complex. The structure of human Notch3 EGF10 was generated *via* SWISS-MODEL and docked against POGLUT2–UDP complex using the ClusPro 2.0 server with default parameters, allowing side-chain flexibility within 9 Å of the binding interface, which generated 91 protein–protein docking models for POGLUT2–UDP–N3EGF10 complex.

Top-ranking models were selected according to cluster scores, weighted scores, and biological relevance, including proper positioning of the C^3^–C^4^ consensus sequence within the POGLUT2’s binding cleft, proximity of EGF-repeat Ser414 (the residue to be modified) to UDP β-phosphate, and the absence of significant steric clashes. The final complex model was validated through extensive functional mutagenesis characterizations (see details in Figs. 3 and 4).

For sequence logo analysis, multiple sequence alignments of EGF repeats were performed using Clustal Omega (https://www.ebi.ac.uk/Tools/msa/clustalo/), and sequence conservation was visualized as sequence logos using WebLogo-3.7.9 (http://weblogo.threeplusone.com/).

### Mutagenesis and enzyme activity measurement

Site-directed mutagenesis of POGLUT2 and EGF-repeat acceptor substrates was performed using a PCR-based overlapping extension method with WT gene sequences as templates. All introduced mutations were verified by direct DNA sequencing. The mutant proteins were expressed and purified following the same procedures as for the WT proteins. GT activity was assayed using the UDP-Glo GT Assay kit (Promega). The standard reaction mixtures (20 μl) contained 10 μM WT or mutant EGF-repeat acceptor substrate, 0.1 μg WT or mutant POGLUT2, and a reaction buffer containing 20 mM Tris–HCl (pH 7.5) and 150 mM NaCl. The reaction was initiated by adding UDP-glucose (Sigma–Aldrich) to a final concentration of 10 μM, followed by incubation at 37 °C for 1 h. After the reaction, an equal volume (20 μl) of UDP-Glo Detection Reagent was added to each reaction mixture and incubated at 25 °C for 1 h. Luminescence was measured using a PHERAstar FS system (BMG LABTECH). All experiments were performed in triplicate. Background control reactions were carried out in the absence of EGF-repeat substrates.

### MST assay

The binding affinities between WT or mutant POGLUT2 and WT or mutant EGF repeats were measured *via* MST assay on a Monolith NT.115 instrument (NanoTemper Technologies). His-tagged POGLUT2 variants were fluorescently labeled with the NT-647 RED dye according to the manufacturer’s instructions. For each binding assay, 200 nM of labeled protein was mixed with an equal volume of serially diluted unlabeled EGF repeats across 16 concentrations in assay buffer (20 mM Hepes [pH 7.5] and 150 mM NaCl) and incubated for 15 min at room temperature. After incubation, the samples were then loaded into MST capillaries (NanoTemper Technologies), and measurements were carried out at 25 °C with 60% LED power and medium MST power. Binding affinities of hN3EGF10, hN3EGF10 F415A with the WT POGLUT2 and mutants (F338A, H353A, and Y270A) were measured following the same procedures. Each assay was repeated at least three times. *K*_*d*_ values were determined using Monolith Affinity Analysis v.2.2.4 software (NanoTemper Technologies) and binding curves were plotted with GraphPad Prism 8.0 (GraphPad Software, Inc).

### Western blots

WT and filamin domain–truncated (ΔFilamin) POGLUT2 constructs were generated and expressed in *P*. *pastoris*, as described above. Following induction, equal volumes of each yeast culture were collected. The culture supernatant was harvested, and the corresponding cell pellets from WT and ΔFilamin samples were lysed under identical conditions. Equal volumes of the supernatant and lysate samples were then separated by 10% SDS-PAGE and transferred onto a nitrocellulose membrane (MilliporeSigma). Membranes were probed with a mouse anti-His_6_ antibody (catalog no.: 68327-1-Ig, Proteintech), followed by incubation with a horseradish peroxidase–conjugated goat anti-mouse IgG secondary antibody (catalog no.: SA00001-1, Proteintech). GAPDH was used as a loading control and detected using a rabbit anti-GAPDH antibody (catalog no.: 10494-1-AP, Proteintech) and a horseradish peroxidase–conjugated goat anti-rabbit IgG secondary antibody (catalog no.: SA00001-2, Proteintech). Protein bands were visualized using the Odyssey imaging system (LI-COR Biosciences).

## Data availability

The coordinate and structure factor of human POGLUT2–UDP binary complex has been deposited at the Protein Data Bank with accession code 9W47.

## Supporting information

This article contains supporting information.

## Conflict of interest

The authors declare that they have no conflicts of interest with the contents of this article.
