# Supplementary material for: Structural basis of EGF-repeat O-glucosylation by the protein O-glucosyltransferase POGLUT2
Source: J Biol Chem. 2026 Mar 9;302(5):111361. doi: 10.1016/j.jbc.2026.111361 (PMC13084666; doi:10.1016/j.jbc.2026.111361)

Supporting Information

Structural basis of EGF-repeat *O*-glucosylation by the protein *O*-glucosyltransferase POGLUT2

Yuying Xia, Xinlin Hu, Zhengkang Hua, Min Zhang, Xuyang Ding, Yunshu Shi, Yan Ke, Jiameng Li, Hongjun Yu

Email: [hongjun_yu@hust.edu.cn](mailto:hongjun_yu@hust.edu.cn)

**This file includes:**

Figure S1 to S4

Table S1

**SUPPLEMENTARY FIGURES**


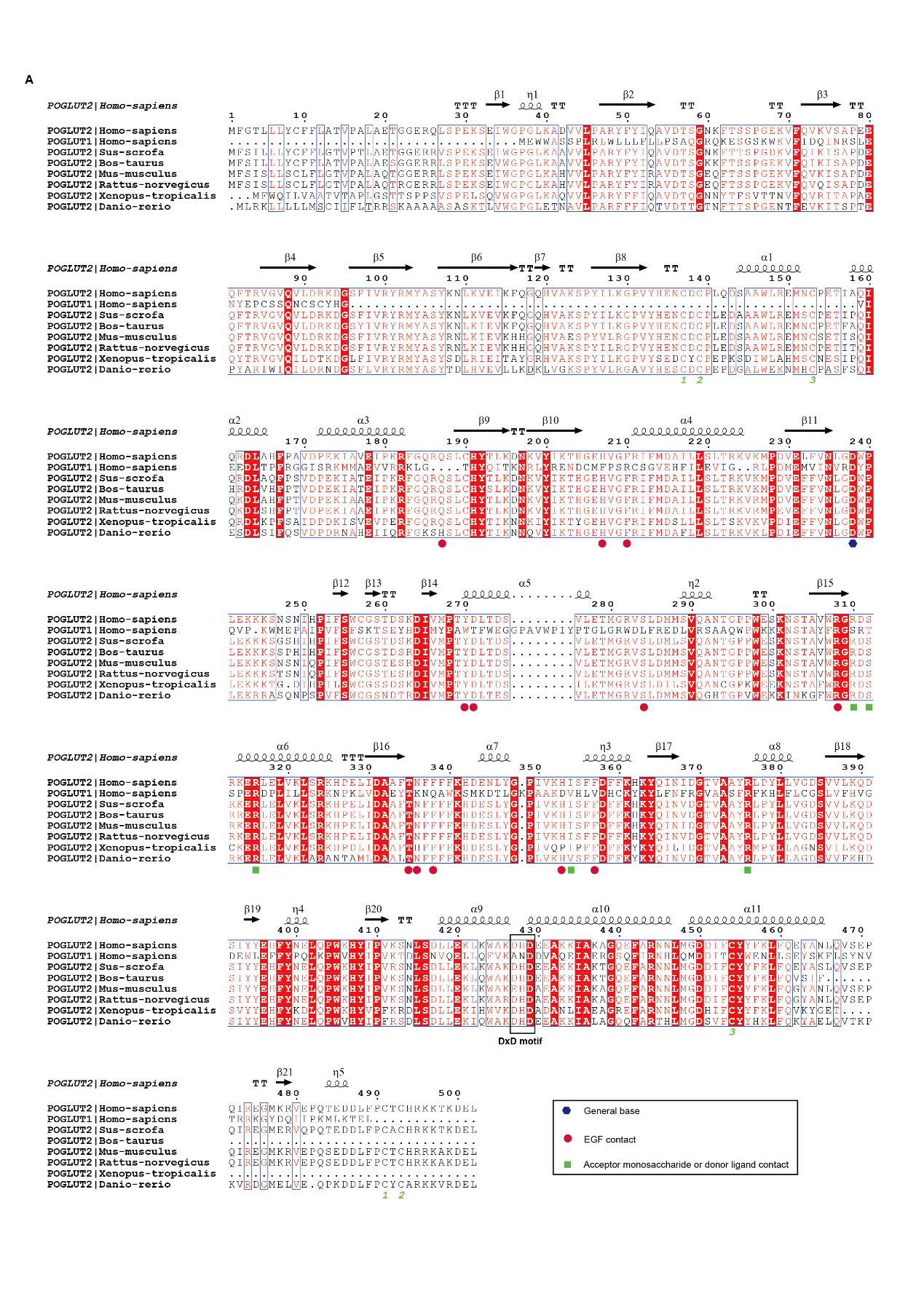


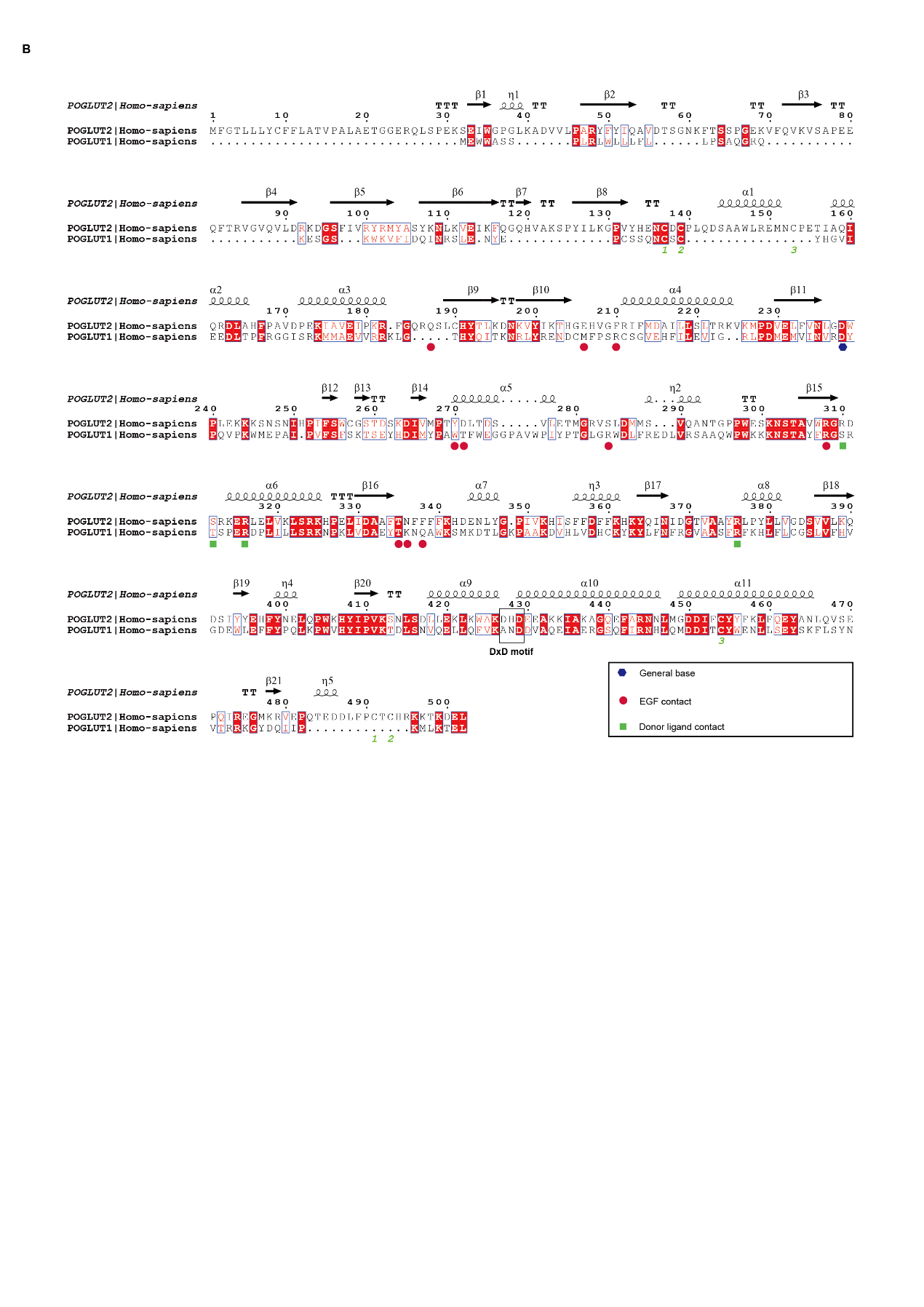


**Supplementary Figure 1. POGLUT2 sequence conservation analysis.**

**A.** Multiple sequence alignment of POGLUT2 across species including *Homo sapiens*, *Sus scrofa*, *Bos taurus*, *Mus musculus*, *Rattus norvegicus, Xenopus tropicalis* and *Danio rerio*.

**B**. Sequence alignment between *Homo sapiens* POGLUT2 and POGLUT1.

Alignments in both (**A**) and (**B**) were generated using Clustal Omega and visualized using ESPript3. Functionally important residues are annotated and labeled below the alignments.


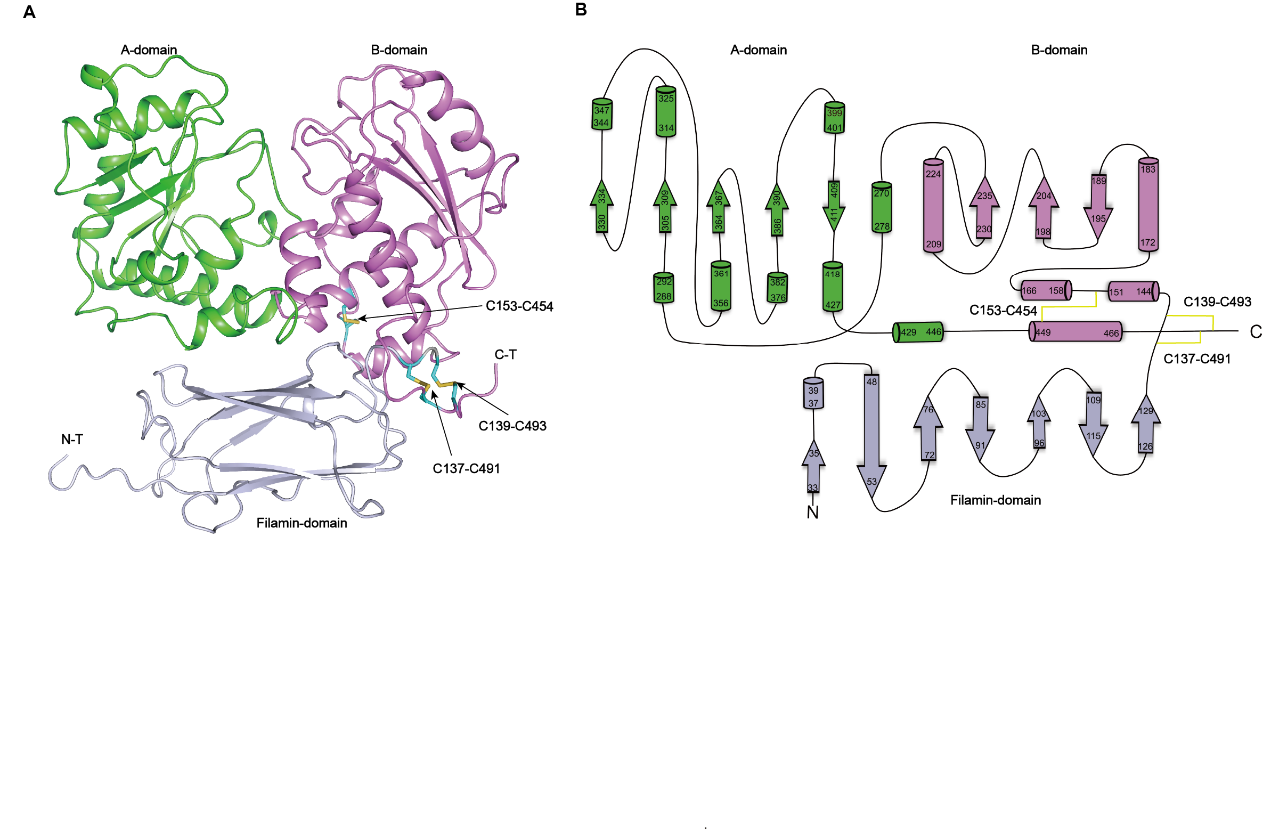


**Supplementary Figure 2. Domain organization of POGLUT2.**

**A.** Domain architecture of POGLUT2. Three domains are shown in cartoon representation: A-domain (green), B-domain (pink), and Filamin-domain (dark grey). Three disulfide bonds (C153-C454, C139-C493, C137-C491) are shown as yellow sticks.

**B.** Topological diagram illustrating the three-domain arrangement of POGLUT2. The three disulfide bonds are depicted with yellow lines.

**
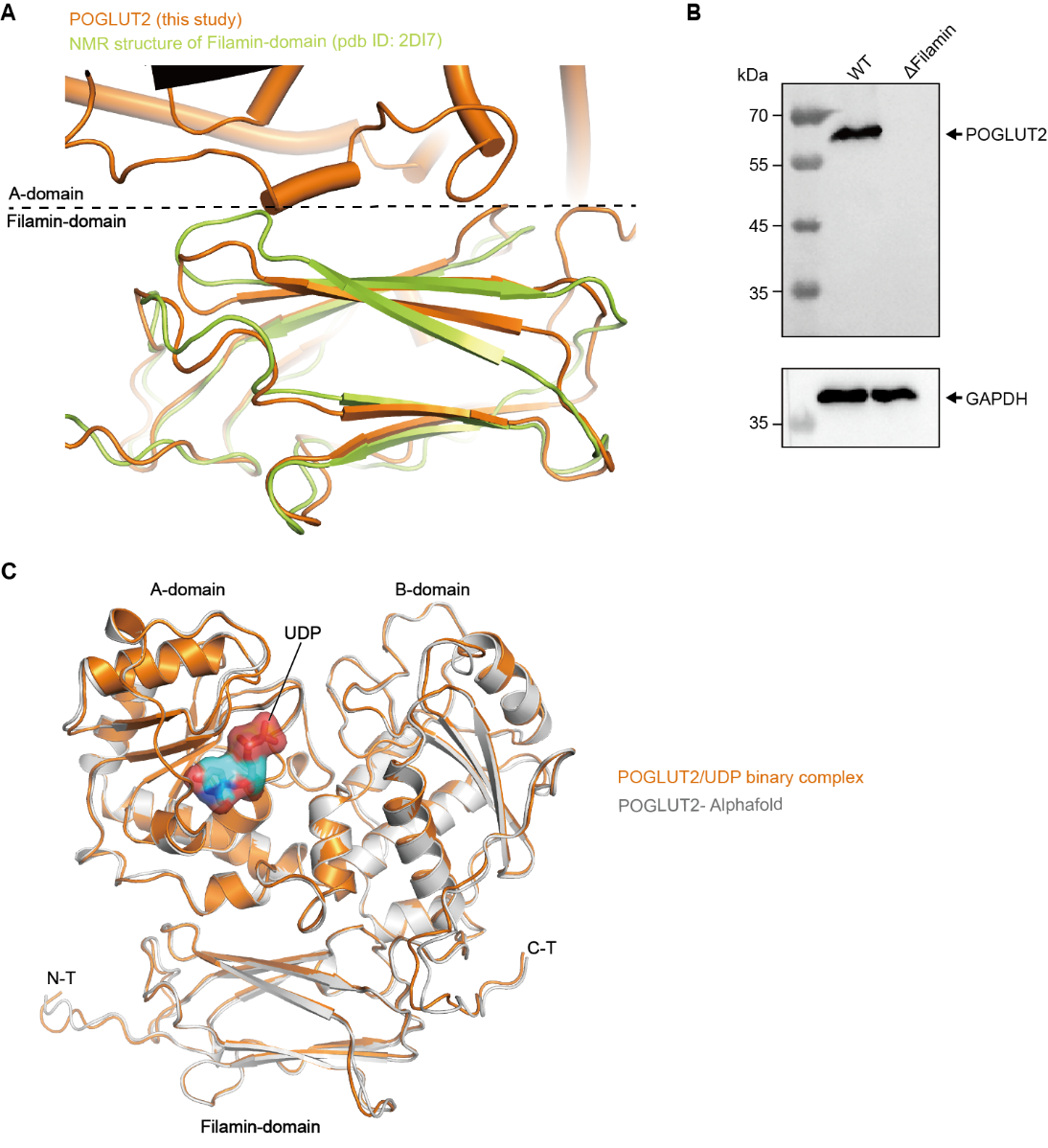
**

**Supplementary Figure 3. Comparison analysis of POGLUT2-related structures.**

**A.** Superposition of POGLUT2 structure (orange; this study) and the NMR structure of the Filamin-domain (green; PDB ID: 2DI7). The dashed line indicates the interface between A-domain and Filamin-domain of POGLUT2.

**B.** Relative expression levels of His_6_-tagged wild-type POGLUT2 (WT) and its Filamin-domain-truncated variant (ΔFilamin), detected in the culture supernatant by western blotting with an anti-His_6_ antibody. GAPDH was used as a loading control.

**C.** Superposition of POGLUT2/UDP binary complex structure (POGLUT2 in orange; UDP labeled) and AlphaFold-predicted structure of apo POGLUT2 (gray).


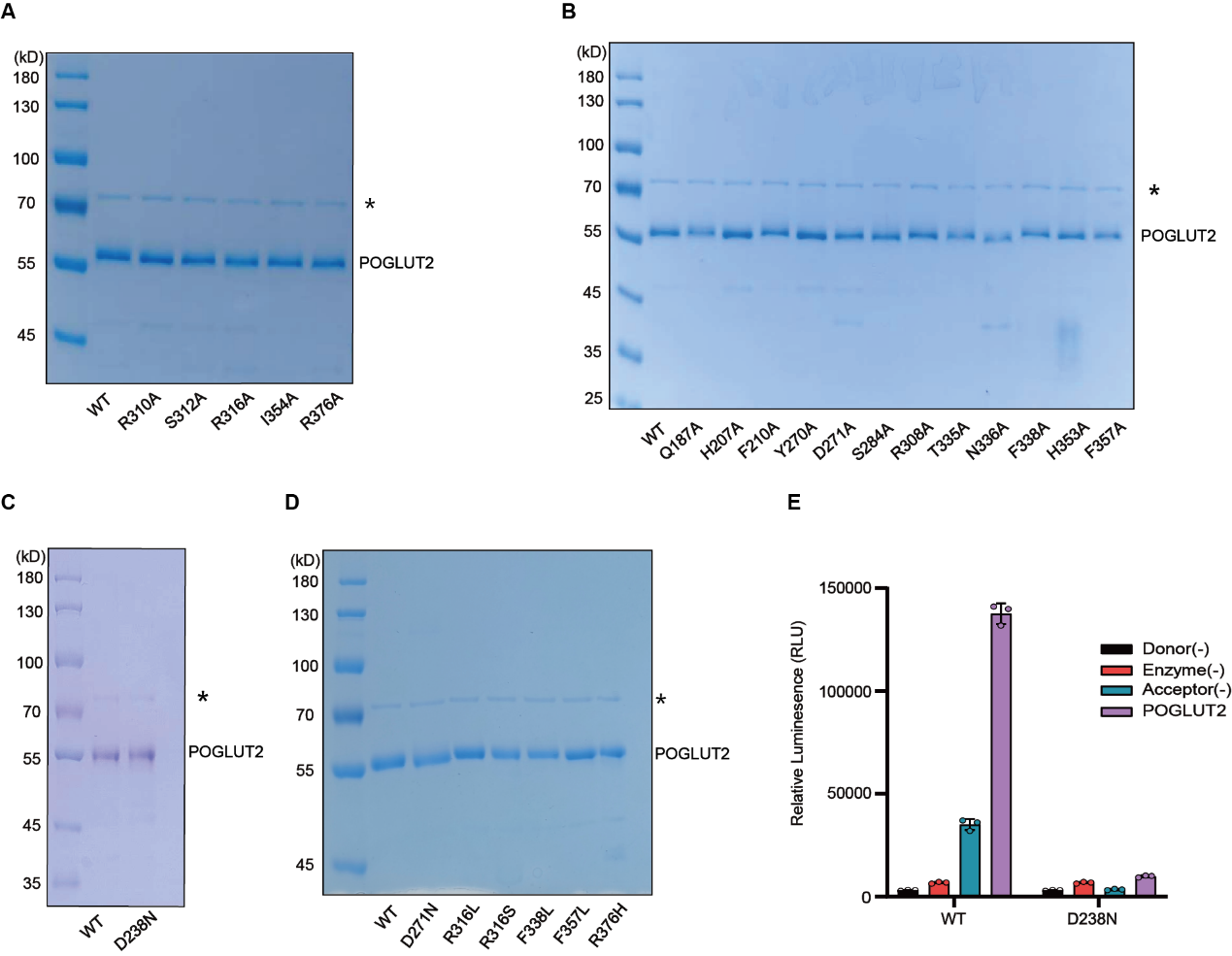


**Supplementary Figure 4. Site-directed mutagenesis of POGLUT2.**

**A-D.** SDS-PAGE analyses of purified wild-type POGLUT2 and its mutants. The purified proteins were detected as a single band with the molecular weight of approximately 58 kDa. One additional band (marked with an asterisk) corresponds to Endo Hf, which was used for N-glycan cleavage.

**E.** Background controls for the activity assay of WT POGLUT2 (left) and POGLUT2 D238N variant. Full reactions containing both enzyme and substrates are shown in purple (POGLUT2). Three negative control conditions were included: donor (–) (donor omission, black); enzyme (–) (enzyme omission, red); acceptor (–) (acceptor omission, cyan). Data represent mean ± SD from three independent assays.

**Supplementary Table 1. Data collection and structure refinement statistics of POGLUT2-UDP complex**


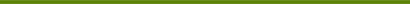


| Structure | POGLUT2-UDP |
| --- | --- |


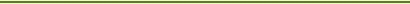


| **Data collection** |  |
| --- | --- |
| Space group | P 1 21 1 |
| Cell dimensions |  |
| *a, b, c* (Å) | 47.13, 63.28, 89.53 |
| α, β, γ (°) | 90.00, 95.96, 90.00 |
| Resolution (Å) | 63.28-1.79 (1.83-1.79) |
| Wavelength (Å) | 0.9791 |
| *R*_merge_ (%) | 6.9 (71.5) |
| *I* / *σI* | 16.2 (3.2) |
| CC_1/2_ (%) | 99.8 (74.7) |
| Completeness (%) | 97.0 (95.4) |
| Number of total reflections  Number of unique reflections | 313744  47885 |
| Redundancy | 6.5 (5.8) |
|  |  |
| **Refinement** |  |
| Resolution (Å) | 29.68-1.79 (1.85-1.79) |
| No. reflections | 47861 |
| *R*_work_*/R*_free_ (%) | 20.16/22.74 |
| No. atoms | 4032 |
| POGLUT2 | 3836 |
| Water | 165 |
| Nucleotide (sugar) | 25 |
| Water, sulfate or Glycerol | 6 |
| *B*-factors (Å²) | 38.51 |
| POGLUT2 | 38.7 |
| Water | 33.3 |
| Nucleotide (sugar) | 46.2 |
| R.m.s. deviations |  |
| Bond lengths (Å) | 0.0081 |
| Bond angles (°) | 1.00 |
| Ramachandran plot (%) |  |
| Favored | 97.63 |
| Allowed | 2.16 |
| Disallowed | 0.22 |


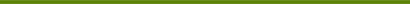

Supplement: Supplementary Material — 1 [file mmc1.docx]
